# Association between continuity of care (COC), healthcare use and costs: what can we learn from claims data? A rapid review

**DOI:** 10.1186/s12913-022-07953-z

**Published:** 2022-05-16

**Authors:** Anna Nicolet, Muaamar Al-Gobari, Clémence Perraudin, Joël Wagner, Isabelle Peytremann-Bridevaux, Joachim Marti

**Affiliations:** 1 Center for Primary Care and Public Health (Unisanté), University of Lausanne, Biopôle 2 SV-A, Route de la Corniche 10, CH-1010 Lausanne, Switzerland; 2 Department of Actuarial Science, Faculty of Business and Economics (HEC), and Swiss Finance Institute, University of Lausanne, Lausanne, Switzerland

**Keywords:** Continuity of care, Claims-based data, Healthcare use, Costs

## Abstract

**Objective:**

To describe how longitudinal continuity of care (COC) is measured using claims-based data and to review its association with healthcare use and costs.

**Research design:**

Rapid review of the literature.

**Methods:**

We searched Medline (PubMed), EMBASE and Cochrane Central, manually checked the references of included studies, and hand-searched websites for potentially additional eligible studies.

**Results:**

We included 46 studies conducted in North America, East Asia and Europe, which used 14 COC indicators. Most reported studies (39/46) showed that higher COC was associated with lower healthcare use and costs. Most studies (37/46) adjusted for possible time bias and discussed causality between the outcomes and COC, or at least acknowledged the lack of it as a limitation.

**Conclusions:**

Whereas a wide range of indicators is used to measure COC in claims-based data, associations between COC and healthcare use and costs were consistent, showing lower healthcare use and costs with higher COC. Results were observed in various population groups from multiple countries and settings. Further research is needed to make stronger causal claims.

**Supplementary Information:**

The online version contains supplementary material available at 10.1186/s12913-022-07953-z.

## Introduction

Continuity of care (COC) is considered a quality indicator and a cornerstone in primary care [1–3]. COC is a multidimensional concept with several core elements - repeated and regular visits to a health professional sustained over time (i.e. longitudinal care); a relationship of trust and responsibility between patients and health professionals; and cooperation and communication of relevant information between providers within and between care settings [4, 5]. In practice, three broad sets of dimensions are used: informational, interpersonal, and longitudinal continuity of care [6–8]. Informational continuity reflects the availability (storage and access) and the way of transmission (verbal, electronic, written) and sharing of clinical information to providers involved in the patient pathway [6, 9, 10]; systems enhancing informational continuity (e.g., Electronic Medical Record, EMR) deserve more attention and may serve to counterbalance the effect of interruptions in continuity of care in some circumstances [9, 11, 12]. Interpersonal continuity reflects the quality of personal relationships between patients and providers. It often focuses on concepts such as personal trust and responsibility [6, 8, 13, 14]. Finally, longitudinal continuity - the focus of this study, is a quantitative assessment of the continuity of individual care trajectories over time. It is typically measured using indices that reflect the concentration (higher proportion of visits to a specific doctor among other doctors imply higher COC), dispersion (higher overall number of doctors visited imply lower COC), density (more frequent sharing of patients among providers implies higher COC), or sequence of doctor visits (whether the same doctor is visited from one time to the next) [15, 16]. Such indicators may reflect the fragmentation of care trajectories, which may be potentially associated with poor coordination, duplication of low-value services, or polypharmacy [17]. In fact, poor COC was found to be negatively associated with a range of patient outcomes including patient satisfaction, avoidable hospitalizations, readmissions, mortality, or increased healthcare costs [8, 18–23]. These negative outcomes affect patients, particularly those with multiple chronic diseases since several healthcare providers are usually involved in their care [18, 22, 24–26]. By contrast, we presume that better continuity can lead to fewer unnecessary repeated diagnostic tests and adverse drug interactions. It might also lead to higher outpatient costs (more regular doctor visits), but lower inpatient costs (fewer hospital admissions), thus resulting in lower overall costs. Consequently, focusing on the effect of COC on healthcare utilization and costs may provide important insights into the potential contribution of COC into healthcare efficiency improvement and unnecessary utilization reduction.

The measurement of COC indices requires rich longitudinal data on individual interactions with healthcare providers, which is often problematic to acquire in countries without national registries. Therefore, we considered claims-based data as a good opportunity to study longitudinal COC as they are routinely collected on a large-scale, are relatively easy to access, and provide the advantages of capturing the patient trajectories and costs across different providers over time. Such data are mainly collected for billing and reimbursement purposes with an implicit financial incentive that ensures their regular and comprehensive collection and management.

Several studies have been performed on COC, its measurement and association with health outcomes [4, 5, 7, 14, 22, 27–29], accounting for a single dimension of COC (e.g., longitudinal) or broader dimensions. None of these studies narrowed the focus on two important components fostering COC for health system effectiveness: the effect on healthcare utilization and costs, and the use of claims data allowing rich and long-term observations (including administrative databases that are comparable to claims data used for provider billing). Despite differences in the structure and content of administrative databases across countries, several of these databases can be used to measure aspects of COC.

The aim of this paper is to review various indicators of longitudinal COC used in claims data, and the evidence on their associations with healthcare use and costs. We will also assess methodological quality of the studies and express recommendations for future studies.

## Materials and methods

We conducted a rapid review [30] of the published peer-reviewed literature, due to time and resources available considerations, knowing that systematic reviews are very resource-intensive. We adhered to PRISMA guidelines as closely as possible and consulted with other literature [31, 32] (protocol registered on PROSPERO [33]), while any derogations and potential biases are reported in the discussion section.

### Literature and information search

We searched Medline (Pubmed), EMBASE, and Cochrane Central from inception up to April 1, 2019 (updated in December 2020). The search strategy (Appendix 1) comprised Medical Subject Heading Terms (MeSH) (e.g., "Continuity of Patient Care" [Mesh]), free text words (e.g., "claims data" [tiab]), Boolean terms (e.g., AND, OR) and truncations (e.g., measur*) where necessary. Beside electronic searches, we manually searched references lists of identified studies, and used the google search engine as well as the google scholar platform to identify additional eligible studies.

### Eligibility of studies

First, primary studies were included if the study author(s) measured continuity of care (COC) and used claims-based data or administrative data for billing purposes. Per protocol, self-reported questionnaires and surveys as well as editorials, conference papers, letters and non-English papers, were excluded. Additionally, we excluded systematic reviews at the first stage but screened them at a later stage to identify primary studies or potential COC indicators. At the final stage, we excluded the studies not investigating associations between COC, healthcare use and/or costs.

### Data items

Our review included primary studies that used COC indicators in the published literature using claims-based data. Out of various possible outcomes, including medication adherence, quality of care, disease/episode incidence, number of chronic conditions and mortality, we focused on healthcare use and costs as dependent variables. Any measure of healthcare use was accepted, such as visits to emergency departments (ED), hospital admissions and re-admissions, likelihood of hospitalization (general or disease-specific), or avoidable/preventable hospitalizations (e.g., hospitalization for ambulatory care-sensitive condition - ACSC). Any measure of healthcare costs was accepted, such as total, inpatient, outpatient care costs, or disease-specific costs. From the included studies, we extracted the following data: study identification information, study design, aim/purpose, source of claims-based data, type of population/setting, sample size, follow-up, COC indicators or indices, period of COC measurements (discrete or over the whole period of follow-up), types of outcomes associated with COC and main findings. One reviewer (MA) was involved in the search and initial selection of the included studies while three reviewers (MA, AN, CP) participated in data extraction and took final decisions on the inclusion/exclusion of eligible studies. We used https://rayyan.qcri.org website for the process of study selection [34]. Discrepancies were resolved upon discussions and consensus.

### Risk of bias (quality) assessment

The quality of the studies and risk of bias were evaluated using the Newcastle-Ottawa Scale (NOS) for all included studies [35]. We used the NOS for cohort studies and the adapted version of NOS for cross-sectional studies [36]. As the issue of causality is not explicitly covered by NOS, we checked whether causality and related biases have been formally considered in the study design or acknowledged as study limitations. There is a potential risk of time bias when the COC measurement period does not precede the outcomes measurement period. In this case, it is unclear whether a change in COC changed the outcomes, or vice versa (reverse causality). In addition, attributing changes in outcomes to changes in COC is challenging due to potential omitted variable bias.

### Data analysis

We reported the median of sample sizes of the included studies and reported direction and statistical significance of the associations (e.g., odds ratio (OR), hazard ratio (HR), beta-coefficients) between COC and healthcare use and costs. Additionally, we categorized the measures of COC based on the concepts they represented (e.g., density, dispersion) and on the frequency of their use in the primary studies. We also considered the timing of COC measurement, as COC measured in discrete consecutive periods (e.g., yearly) may not reflect the same type of continuity as measured over the full observation period (e.g., 2 or 4 years).

## Results

### Literature search yield

**Fig. 1:**
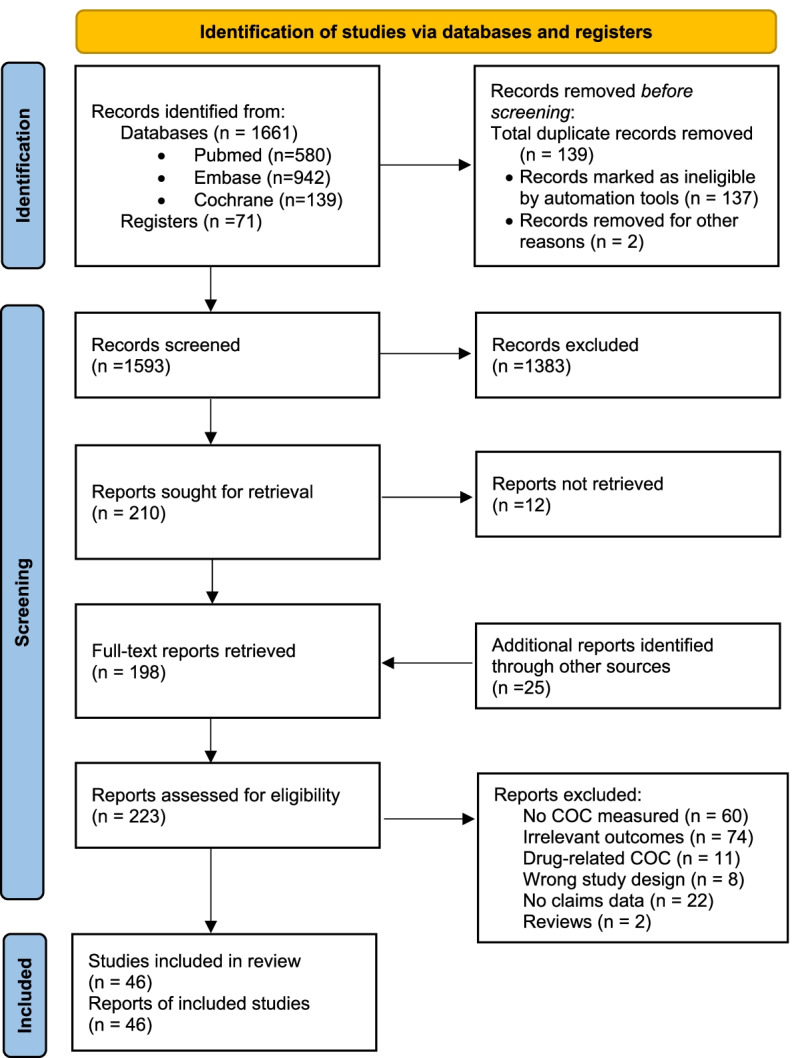
Flow chart for search result

Our electronic and hand searches identified 1,593 potentially relevant studies after removal of duplicates. We excluded 1,383 records based on title/abstract screening. After that, we searched for full texts for the remaining records and manually added potentially eligible studies from the references of retrieved full texts. More studies (*N* = 12) were excluded due to unavailability of the full texts. Overall, 223 full-text studies were screened against our inclusion criteria, 177 of them were excluded for different reasons, finally arriving at 46 primary studies (Figure 1).

### Types of study designs, populations and settings

All studies except six [20, 37–41] were retrospective cohorts; most were conducted in East Asia (*n*=23) and North America (*n*=20) (Table 1). The sample size of included studies ranged from 252 to 3,276,635 subjects with a median of 18,113. The vast majority of the studies targeted adults (*n*=39). Whereas 14 reported on patients with diabetes mellitus, 11 reported on patients with chronic obstructive pulmonary disease (COPD) or asthma, five on heart failure patients, three on hypertensive patients, and the remaining studies were conducted on other patient groups or the general population. All studies were assessed as medium to high quality with all studies showing a rating higher than 6/9 (for cohort studies) and 7/10 (for cross-sectional). Also, all studies investigated associations, adjusting for a wide range of covariates, getting at least one score (out of two maximum) for comparability. Out of 46 studies, causality issues were discussed in 18 studies, while only two studies [42, 43] made claims of a causal effect of COC on the outcomes. Furthermore, 19 studies acknowledged or accounted for time bias only (related to causality issue), which is a necessary but not sufficient condition for making claims of causality, while nine studies never addressed adjustment for time bias or discussed causality issue.

**Table 1:** Main characteristics of included studies

**Author year; country**	**Study design**	**Main aim of the study**	**Type and source of data**	**Sample size (N) and population**	**Follow-up**	**COC indicators**	**NOS score**	**COC measurement period**	**Causality discussed**	**Outcomes associated with COC**	**Main results of interest**
Bazemore 2018 ; USA [37]	CS	To examine the relationship between physician-level continuity and healthcare expenditures and hospitalizations	US Medicare claims data; Medicare Quality Payment Program	1,448,952 Medicare beneficiaries	2011	UPC; COCI; MMCI; HI	9/10	Unclear	No	Healthcare expenditures and hospitalizations	Higher COC was associated with lower total expenditures (β = –0.151; CI: –0.186 –0.116) and hospitalization rates (OR=0.839, CI:0.787-0.893)
Blozik 2020; Switzerland [44]	RC	To evaluate continuity of care in Swiss cancer patients based on routine data of mandatory health insurance using four established continuity scales	Helsana health insurance group claims data	23ʹ515 patients with incident use of antineoplastics	2014-2017	UPC; MMCI; COCI; SECON; composite	9/9	Single year	Partly, reversed causality accounted in design	Costs, death, and hospitali-zation by type of consul-tations: all, only with GP, with specialists, ambulatory	Higher COC was associated with lower costs for consultations with GP (β=-0.035, *p*<0.01 for COCI; β=-0.092, *p*<0.001 for SECON) and mixed for ambulatory consultations (β=0.041, *p*<0.001 for COCI; β=-0.114, *p*<0.001 for SECON), while higher costs for all consultation (β=0.087, *p*<0.001 for COCI; β=0.033, *p*<0.01 for SECON) and mixed results for the costs for consultations with specialists (β=0.106, *p*<0.001 for COCI; β=-0.072, *p*<0.001 for SECON). For hospitalizations the results are mixed: higher COC is associated with higher OR of hospitalizations for all consultations (OR=1.77, *p*<0.001 for COCI; OR=1.32, *p*<0.01 for SECON), lower OR for consultations with GP only (insignificant), higher OR for consultations with specialists (insignificant) and mixed results for ambulatory consultations (significant for UPC and MMCI only: OR=0.74, *p*<0.01 identically for both)
Chen 2011 ; Taiwan [45]	RC	To examine the effects of COC on healthcare utilization and expenses	NHI claims data set	48,107 DM patients	2000-2006	COCI	9/9	Each year of follow-up	Partly, acknowledged in limitations and applied in the design	Healthcare utilization, ED visits and healthcare expenses	High COC compared to low COC was associated with less hospitalizations (OR=0.26, CI:0.25-0.27) and ED visits(OR=0.34, CI:0.33-0.36) and lower healthcare expenses (pharmaceutical expenses: β= –0.14, *P* <.001, total diabetes-related expenses: β= –0.53, *P* <.001)
Chen 2019; Taiwan [46]	RC	To examine the effects of COC on the utilization of follow-up services and outcome of breast cancer patients (stages I–III) in the post-treatment phase of care	Taiwan Cancer Registry, National Healthcare Insurance Database (NHIRD) and the National Register of Deaths	18,031 patients newly diagnosed with breast cancer (Stage I, II, or III)	4 periods of 2 years in 2002-2007	COCI	8/9	Each year for 4 follow-up periods	Partly, reversed causality accounted in design	Hospital admission, emergency department visit	Higher oncology COCI was associated with a lower likelihood of hospitalization (OR = 0.78, 95% CI: 0.71–0.85) and emergency department use (OR = 0.88, 95% CI: 0.82–0.95). A higher PCP COCI was also associated with a lower likelihood of hospitalization (OR = 0.77, 95% CI: 0.70–0.85) and emergency department use (OR = 0.75, 95% CI: 0.68–0.82).
Chen 2020a; Taiwan [47]	RC	To examine and provide quantitative evidence of the effects of COC on end-of-life care among patients with ESRD	National Health Insurance Research Database of Taiwan	29,095 elderly patients with end-stage renal disease	2005-2013	COCI	6/9	6-12 months before death	Partly, reversed causality accounted in design	Healthcare expenditures admissions to the Intensive Care Unit (ICU), visits to the Emergency Room	Better COC would result in lower cumulative expenditures: 1% increase in COCI was associated with an 8%, CI: 7–9% and a 6%, CI: 4%–7% reduction in the total health costs in the 6 and 3 months before death. Patients with perfect provider COCI (provider COCI=1) had lower utilization of ICU: OR=0.78, CI: 0.67-0.90. Patients with perfect site COCI had an OR of 0.74 (CI:0.67-0.83) in the utilization of the ER
Chen 2020b; Taiwan [48]	RC	To examine the relationship between COC and care coordination, simultaneously evaluate the effects of COC and care coordination on healthcare outcomes, and investigate whether these effects vary across study subjects with different levels of comorbidity	Longitudinal Cohort of Diabetes Patients claims data sets constructed by the National Health Research Institute in Taiwan	57,965 patients aged 18 years or older who were newly diagnosed with diabetes	2007-2011	COCI, Care density	8/9	Each year of 3 years follow-up	Partly, reversed causality accounted in design and discussed in limitations	Hospital admission for diabetes or cardiovascular/cerebrovascular conditions	Multi-morbid patients with high- or median-COCI were less likely to be hospitalized for diabetes-related conditions than those in the low-continuity group (OR=0.86, CI: 0.77- 0.96) and (OR=0.90, CI: 0.83-0.98), respectively. For care coordination, the patients in the high- or median-care-density groups were less likely to be hospitalized for diabetes-related conditions than the patients in the low-care-density group (OR=0.84, CI: 0.76-0.93, and OR=0.91, CI:0.84-0.99).
Cho 2015 ; Korea [38]	CS	To analyze the association between continuity of ambulatory care and hospital admission and determine which continuity index has the best explanatory ability for hospital admission	National Health Insurance Sample (NHIS)	54,458 participants with type 2 DM	2009	UPC; COCI; SECON; ICOC	10/10	Single year	No	Hospital admission	Low COC was associated with higher probability to be hospitalized (OR=2.44, CI: 2.17-2.75)
Cho 2016 ; Korea [49]	RC	To assess the effects of competition among healthcare providers with regard to COC, and the association between COC and hospital admissions	Korean universal health insurance program	9,997 Asthmatic children	2009-2013	UPC	7/9	Each year of follow-up	No	Hospital admissions	Lower COC (below mean) is associated with increased hospital admission (OR=2.72, CI:2.14-3.46)
Christakis 2001a ; USA [50]	RC	To search if COC is associated with better quality of care	Administrative claims data; Washington State Medicaid	252 Medicaid children with DM (<18 years old)	1997	COCI	7/9	Single year	No	Hospitalization (inpatient DKA)	High and medium COC was associated with reduced risk of hospitalization for DKA (OR=0.14, CI:0.03-0.67 and OR=0.22, CI:0.05-0.87, respectively)
Christakis 2001b ; USA [51]	RC	To examine the association between COC and ED visits and hospitalization	Claims data of Group Health Cooperative	46,097 children	1993-1998	COCI	9/9	Unclear	Yes, addressed in the design and discussed in limitations	ED use and hospitalization	Lower COC was associated with higher risk of ED visits (HR=1.58, CI:1.49-1.66), ED visits for asthma (HR=1.13, CI:0.82-1.60), hospitalization (HR=1.54, CI:1.33-1.75) and asthma-related hospitalization (HR=1.79, CI:1.21-2.56)
Christakis 1999 ; USA [52]	RC	To assess whether greater COC was associated with lower ED use	Outpatient teaching clinic at Children’s Hospital and Regional Medical Center, Seattle	785 Medicaid children (≤19 years)	1993-1997	COCI	7/9	Unclear	Partly, reverse causality was accounted for in the design	ED use	Higher COC was associated with an decreased ED utilization (HR=0.65, CI:0.50-0.80)
CHU 2012 ; Taiwan [53]	RC	To examine the association between COC and potentially inappropriate medication use and indirectly with healthcare outcomes and expenses	Longitudinal Health Insurance Database	51,804 patients (≥65 years)	2004-2009	COCI	9/9	Each year of follow-up	Partly, omitted variables bias was addressed in the study design	Healthcare outcomes and expenses	Higher COC was associated with lower risk of hospitalization (OR=0.37, CI:0.36-0.38) and ED visits (OR: 0.44, CI:0.43-0.45), and lower total healthcare expenses (β=-0.40, *p*<0.001)
Gill 2000 ; USA [39]	CS	To examine the association of COC with ED visits	Delaware Medicaid database	11,474 subjects	1993-1994	MMCI	9/10	Single year	Partly, reverse causality acknowledged as limitation	ED visits	High provider continuity is associated with lower single ED visit (OR=0.82, CI:0.70-0.95) and multiple ED visits (OR=0.65, CI:0.56-0.76)
Hong 2013 ; Korea [54]	RC	To examine the effects of continuity of ambulatory care on health outcomes	Korean National Health Insurance Program	68,469 patients; type 2 DM	4 years	COCI	9/9	Unclear	Yes, addressed in the design and discussed in limitations	Hospitalization and healthcare costs	Lower COCI (compared to perfect COC of 1) was associated with increased risk of all-cause hospitalization (OR: 1.37, CI: 1.28-1.47) and healthcare costs (β= 0.037, *P* < 0.001)
Hong 2010; Korea [55]	RC	To assess association of COC with health outcomes	Korea National Health Insurance Claims Database	268,220 DM patients; 858,927 hypertension;129,550 asthma; 131,512 COPD (age: 65 to 84)	2002-2006	COCI	8/9	First 3 years	Yes, addressed in the design and discussed in limitations	Hospitalization, ED visits, healthcare costs	Low COC was associated with increased risk of hospitalization for patients with DM/ hypertension/ asthma/COPD (OR=1.47/ 1.31/2.07/1.99, CI:1.41-1.52/1.28-1.35/1.92-2.23/1.86-2.13, respectively) or ED visits (OR=1.41/ 1.45/2.25/1.77, CI:1.27-1.56/1.34-1.57/1.87-2.70/1.45-2.17, respectively) and increased healthcare costs (β= 0.130/0.116/0.025/0.123, respectively, *P* < 0.001 for all)
Huang 2016; Taiwan [56]	RC	To examine whether continuity of ambulatory care can lower asthma-specific ED utilization	Taiwan National Health Insurance Dataset	29,277 children; asthma patients (age 0 – 17 years)	2006-2009	COCI	9/9	Unclear	Yes, addressed in the design and in limitations	ED utilization	Low continuity of ambulatory care was associated with increased asthma-specific ED utilization (OR=1.38, CI:1.21-1.58)
Hussey 2014; USA [18]	RC	To measure the association of COC with costs, rates of hospitalizations, emergency department visits, and complications for Medicare beneficiaries with chronic disease	US Medicare claims files for a 5% random sample of	241,722 Medicare beneficiaries (CHF: 53,488; COPD: 76,520; DM: 166,654) [age > 65 years]	2008-2009	COCI	8/9	Unclear	Yes, discussed in limitations	Hospitalizations, ED visits, complications, and costs of care	Higher levels of COC among patients with CHF/COPD/DM was associated with lower rates of hospitalization (OR=0.94/0.95/0.95, CI:0.93-0.95/0.94-0.96/0.95-0.96, respectively) and ED visits (OR=0.92/0.93/0.94, CI:0.91-0.92/0.92-0.93/0.93-0.94, respectively), and lower episode costs by 4.7%/6.3%/5.1%, CI:4.4%-5.0%/6.0%-6.5%/5.0%-5.2%, respectively
Jung 2018; Korea [57]	RC	To determine the association between COC and health outcome	Data from the Korea Health Insurance Review and Evaluation Service (HIRA)	311,949 outpatients with knee osteoarthritis	2014	MFPC; MMCI; COCI	7/9	9 months before hospitalizations	Yes, discussed in limitations	Hospitalization and medical expenses	Low COC was associated with increased risk of hospitalization (RR=27.17, CI:3.09-3.51) and medical expenses (β=0.677, *P*<0.001)
Kao 2017; Taiwan [58]	RC	To investigate the relationship between COC and the risk of avoidable hospitalizations	Taiwan’s National Health Insurance claim data	3,356 Elderly asthma patients	2 years/ 2004-2013	COCI	9/9	Single year	Partly, reverse causality is accounted in the design	Avoidable hospitalization	Low continuity was associated with increased risk of avoidable hospitalization (HR=2.68, CI:1.55-4.63)
Kao 2017; Taiwan [58]	RC	To investigate whether high continuity of ambulatory asthma care reduces asthma-related ED visits	Taiwan Health Insurance claims Database 2010	3,395 Elderly asthma patients	2 years/ 2004-2013/	COCI	9/9	Single year	Partly, reverse causality is accounted in the design and limitations	Asthma-related ED visit	Low COC was associated with increased risk of ED visits (HR=2.11, CI:1.37-3.25)
Kao 2019; Taiwan [27]	RC	To investigate associations between COC and ED visits and hospitalization for COPD or asthma	Taiwan National Health Insurance research database	1141 asthma-COPD overlap patients aged ≥65 years	2 years/2004-2013	COCI	8/9	Single year	Partly, reverse causality is accounted in the design	Hospital admission for COPD or asthma, ED visits	The risk of hospital admissions for COPD or asthma for patients in the low and medium COC group was significantly higher than for those in the high COC group (aHR, 1.80; CI: 1.03–3.13; aHR, 1.72; CI: 1.04–2.83, respectively), which is true for ED visits: low COC group (aHR, 2.80; CI: 1.45–5.38); medium COC group (aHR, 2.69; CI: 1.47–4.93).
Kim 2016; Korea [59]	RC	To examine the association of COC with complications health outcomes	Korean National Health Insurance Service (NHIS).	715,053 hypertensive patients	2007-2011	MFPC; MMCI; COCI	9/9	2 years before index event (outcomes one year later)	Partly, reverse causality accounted in the design	Hospitalization, ED visits, complications	Lower COC was associated with increased hospitalization (OR=1.25, CI:1.14-1.36), ED visits (OR=1.38, CI:1.13-1.70) and complications (OR=1.08, CI:1.02-1.13)
Knight 2009; Canada [60]	RC	To investigate the relationship between continuity of family physician care and inpatient hospitalizations	Data from the Medical Care Plan (MCP) physician claims database and the Clinical Database Management System (CDMS)	1,143 elderly DM patients (age ≥ 65 years)	3 years /1998-1999	COCI; UPC; SECON	7/9	Over 3 years after the index event	No	Inpatient hospitalizations	Higher continuity of family physician care was associated with reduced hospitalizations using UPC (OR=0.82, CI:0.68-0.98), COCI (OR=0.82, CI:0.69-0.97), and SECON (OR=0.75, CI:0.61-0.91)
Lai 2016 ; Taiwan [61]	RC	To evaluate the COC and PIM effects	Taiwan Health insurance database	823 DM patients with heart failure (age≥ 65 years)	2005-2010	COCI	8/9	Each year of follow-up	Yes, discussed in limitations	Hospital admissions and ED visits	Higher COC was associated with lower hospital admissions (OR=0.07, CI:0.05-0.10) and ED visits (OR=0.10, CI:0.07-0.13)
Lei 2020a ; USA [42]	RC	To estimate the causal impact of COC on hospitalizations and different reasons for hospitalization	aggregate Veterans Health Administration and Medicare data at the veteran level using Veterans Affairs’	105,528 community-dwelling older veterans with dementia	2014-2015	COCI	9/9	Single year	Yes, accounted in the design and in limitations	Probability of hospitalization	0.1 higher COC resulted in 2.4% (CI: 0.5%–4.4%) lower probability of hospitalization for all causes. COC was not associated with hospitalizations for ACSC. 0.1 higher COC resulted in 3.8% (CI: 2.1%–5.4%) lower probability of hospitalization for neuropsychiatric diseases/disorders.
Lei 2020b ; USA [43]	RC	To estimate the causal impact of COC on total, institutional, and noninstitutional cost among	aggregate Veterans Health Administration and Medicare data at the veteran level using Veterans Affairs’	102 073 community-dwelling older veterans with dementia	2014-2015	COCI	9/9	Single year	Yes, accounted in the design and in limitations	Total costs and costs categories (acute, long-term care costs, ED costs)	0.1 higher BBC resulted in $4045 (CI, $2171-$5919) lower total cost. 0.1 higher COC resulted in $1597 (CI, $688-$2506) lower acute inpatient cost, $119 ( CI, $64-$174) lower ED cost, $4368 (CI, $643-$8093) lower long-stay nursing home cost, $402 (CI, $113-$691) higher medical LTC cost, and $764 (CI, $460-$1067) higher social LTC cost
Li 2019 ; Taiwan [62]	RC	To investigate whether COC is associated with healthcare outcomes and medical care use	Taiwanese National Health Insurance database	4,007 patients with newly diagnosed diabetes	2010-2012	COCI, UPC	7/9	Over 3 years	Yes, discussed in limitations	number of hospital admissions, length of hospital stays, and number of ED visits	The high COCI and UPC groups had significantly lower probabilities of adverse outcomes: probability of hospital admissions (adjusted OR=0.623, CI: 0.543-0.716), probability of ED visits (adjusted OR=0.650, CI:0.570-0.741). high COCI group had a significantly lower incidence rate ratio (IRR) for the number of hospitalizations (IRR=0.75, CI: 0.67-0.83), length of hospitalizations (IRR=0.61, CI:0.52-0.72), and number of ED visits(IRR=0.68, CI: 0.62-0.75)
Lin 2017; Taiwan [63]	RC	To examine the effects of high COC on the risk of avoidable hospitalizations	Longitudinal Health Insurance Database from Taiwan National Health Research Institute	2,199 COPD patients	2005	COCI	8/9	Short term (1 year); long term (2 years)	Yes, accounted in the design	COPD-related avoidable hospitalization	Short-term COC: medium and low COC were associated with increased risk of avoidable hospitalizations, although significant for medium group only (OR=1.89, CI: 1.07-3.33). Long-term COC: medium and low COC were associated with increased risk of avoidable hospitalizations (OR=1.98, CI:1.0-3.94 and OR=2.03, CI:1.05-3.94, respectively)
Lin 2015; Taiwan [64]	RC	To examine the association of COC with the risk of future hospitalization	Longitudinal Health Insurance Database from Taiwan National Health Research Institute	3,015 COPD patients	2005	COCI	9/9	Over 2 years	Yes, discussed in limitations	COPD-related avoidable hospital admission	Lower COC was associated with a higher likelihood of COPD-related avoidable hospitalization (OR=2.29, CI:1.26-4.15)
Lin 2010; Taiwan [65]	RC	To examine the association of discontinuity of care with the risk of hospitalization	Data from Taiwan National Health Research Institute (NHRI).	6,476 DM patients	1997-2002	UPC	9/9	Over follow-up period	Yes, discussed in limitations	Diabetes-related- admission	Lower COC was associated with higher risk of short-term (OR=1.124, CI: 0.547-02.310) and long-term (OR=1.336, CI:1.019-1.728) ACSC admissions.
Madison Hyer 2020; USA [66]	RC	To characterize the impact of continuity of care on perioperative outcomes, as well as on cost of care, among Medicare beneficiaries undergoing hepatopancreatic resection	Medicare claims data.	25,698 Medicare beneficiaries who underwent a hepatopan-creatic surgical procedure	2013-2017	COCI	7/9	Single year	Partly, reversed causality accounted in design	Total costs of surgery, incidence of complications, length of stay, 30- and 90-day readmission and mortality	Among patients undergoing hepatic resection, an increase in COC of 0.2 was associated with decreased costs of 5.1% (CI: -6.3% to -3.8%) compared with a decrease of 2.5% (CI: -3.7% to -1.2%) among pancreatic resection patients. Higher COC was associated with lower 30-day readmission rate(hepatic:COC1st quartile: 13.5% versus COC4th quartile: 11.7%; pancreatic COC1st quartile: 18.6% versus COC4th quartile 16.3%) and lower 90-day readmission rate (hepatic: COC1st quartile: 20.8% versus COC4th quartile: 18.4%; pancreatic COC1st quartile: 27.1% versus COC4th quartile 24.1%)(both *P* <.05).
Mainous III 1998; USA [67]	RC	To examine the association of (site and clinician) COC with the risk of future hospitalization	Delaware Medicaid Patients	13,495 patients; ≥ 3 visits to ambulatory care (age: 0- 65 years)	1993-1995	UPC, Site Index, Clinician index	7/9	Single year	Partly, reversed causality accounted in design	hospitalizations	High COC with a clinician had lower odds of hospitalization (OR=0.75, CI: 0.66-0.87) than high site/low clinician COC. High site/low clinician COC group was not significantly different from low site/low clinician COC (OR=0.93, CI: 0.80-1.08)
Menec 2005; Canada [40]	CS	To examine the association of COC with preventive healthcare and ED use	Administrative data; physician claims data and the Manitoba Immunization Monitoring System (MIMS) database. The Population Registry; Canada Census	536,893 subjects; ≥1 physician contact	1998-1999	Proportion of total visits to family physicians (FPs) made to the same FP - majority-of-care rule	7/10	Over follow-up period	Yes, discussed in limitations	ED visits	Higher COC (>75%) was associated with reduced ED use among children (OR=0.85, CI: 0.78–0.92) and adults (OR=0.85, CI: 0.80–0.90)
Menec 2006; Canada [68]	RC	To examine the relationship between COC and hospitalizations	Survey data linked to administrative physician billing data	1863 subjects (age ≥ 67 years)	1990-1991; 1996-1997	Proportion of total visits to family physicians (FPs) made to the same FP - majority-of-care rule	8/9	Over 2 two-year periods (of follow-up)	No	Hospitalizations	High COC was associated with reduced odds of being hospitalized for ACSC (OR=0.67, CI:0.51-0.90) but not with hospitalizations for all conditions (OR=0.83, CI:0.67-1.01)
Nam 2016; Korea [69]	RC	To examine the association between time-dependent COC and recurrent hospital admissions	Korean National Health Insurance Claims Database (KNHI)	34,607 participants; hypertensive patients	2011–2013	COCI	9/9	over 2011-2012 (single year)	Partly, reversed causality accounted in design	Hospital admissions	Lower COC was associated with a higher risk of hospital admission (HR=1.42, CI: 1.10-1.83)
Nyweide 2013; USA [70]	RC	To examine the relationship between COC and the risk of preventable hospitalization	Claims data FFS Medicare beneficiaries	3,276,635 subjects (age > 65 years	2007-2010	COCI; UPC	8/9	cumulatively each succeeding month until the occurrence of the event	Yes, discussed in limitations	Preventable hospital admissions	Higher continuity of ambulatory care was associated with a lower rate of preventable hospitalization (HR=0.98, CI:0.98-0.99)
Pennap 2020.; USA [71]	RC	To assess the patient-provider continuity of care (CoC) and compare the risk of psychiatric ED visits or hospitalization according to the CoC level.	Medicaid administrative claims data	38,825 individuals, 3–16-year old with a first psychiatric diagnosis between 2009 and 2013	2007-2014	Alpha Index	8/9	Over 2 years	Partly, reversed causality accounted in design	The risk of psychiatric ED visits or hospitalization	The odds of ED visits were higher among youths with low CoC (OR=1.27; CI: 1.13–1.41)or moderate CoC (OR=1.14;CI: 1.02–1.27) compared with those with high CoC Greater odds of psychiatric hospitalization related to low (OR= 1.17; CI: 1.06–1.29) or moderate CoC (OR=1.15; CI: 1.03–1.27) compared with high CoC.
Pollack 2013; USA [41]	CS	To examine the association between patients’ care density and their healthcare costs	Data from 5 large commercial insurance plans	9,596 patients (CHF); 52,668 patients (DM) [age ≥ 40 years]	2009	Care density	7/10	Between baseline and interview time (≥ 1 year apart)	No	Healthcare costs	Patients treated by sets of physicians who shared high numbers of patients (higher care density) tended to have lower costs (total: lower by 3’310$, *p*<0.001, inpatient: 2’563$, *p*=0.001) and rates of hospitalizations (83.4% of hospitalization in low density group, *p*<0.001).
Pollack 2015; USA [72]	RC	To examine if care density is associated with measures of quality	Data from 3 large commercial insurance plans	31,675 (CHF) ; 78,530 (COPD); 240,378 (DM) [age ≥ 40 years]	2008-2009	Care density	7/9	Over follow-up period	Partly, reversed causality accounted in design	30-day readmission, quality indicators	Higher care density was associated with reduced rates of 30-day readmissions (OR=0.68, CI:0.48-0.97)
Reddy 2018; USA [73]	RC	To examine the association of team-based care and COC on high-cost healthcare utilization	Medicare claims data for Veterans Affairs (VA)	1,160,365 patients	2012-2013	UPC	8/9	Single year	Yes, discussed in limitations	ACSC hospitalizations, ED	Increasing COC by 10 percentage points was associated with lower hospitalizations (-4.5, CI [-5.3;-3.7]) and ACSC hospitalizations (-3.2, CI [-3.4;-2.9], but not significantly with ED visits (2.6, CI [-0.2; 5.4]).
Romaire 2014; USA [20]	CS	To examine the effect of COC among beneficiaries who primarily see a PCP and those who primarily see a specialist	Medicare FFS claims data	613,471 Medicare beneficiaries	2007-2009	Predominant Provider; UPC; COCI	10/10	Single year	Yes, discussed in limitations	All-cause hospitalizations, ACSC, all-cause ED visits, and ACSC ED visits, expenditures paid by Medicare	Regardless of specialty type of the predominant provider, higher continuity was associated with lower rates of all cause hospitalization (PCP: IRR=0.91, CI:0.90-0.93, Specialist: IRR=0.91, CI: 0.88-0.95), all-cause ED (PCP: IRR=0.85, CI:1.05-1.11, Specialist: IRR=0.85, CI: 0.83-0.88) and ACSC ED use (PCP: IRR=0.90, CI:0.89-0.92, Specialist: IRR=0.85, CI: 0.82-0.88), and lower expenditures for these services and total costs (PCP: β=-0.158, Specialist: β=-0.131, *p*<0.0001).
Swanson 2018; Germany+Norway [74]	RC	To compare a social health insurance country (Germany) and a national health service country with gatekeeping and patient lists (Norway) on continuity of primary care for patients in terms of GP visits before and after their first hospitalization and compared the effect of COC on 30-day and one-year hospital readmission rates following hospital discharge	German insurance claims data and linked Norwegian national register data; the database for control and payment of reimbursements to health service providers (KUHR), the cause of death register (DÅR) and the GP database (Fastlegedatabasen)	6,373 (Germany); 13,507 (Norway); COPD patients	2009-2014	COCI; UPC; SECON	8/9	Over 2 years before index event and 1 year after	Partly, reverse causality accounted in the design and limitations	Hospital readmissions within 30 and 365 days	Higher GP continuity in primary care was associated with reductions in hospital readmissions, regardless of systems’ set up and COC indices used. Germany 365 day readmission: COCI: OR=0.958, CI: 0.945-0.971, UPC: 0.951, CI: 0.934-0.969, SECON: OR=0.976, CI: 0.962-0.989. Norway 65 day readmission: COCI: OR=0.855, CI: 0.847-0.863, UPC: 0.808, CI: 0.799-0.817, SECON: OR=0.865, CI: 0.857-0.873.
Vogt 2016 ; Germany [75]	RC	To investigate the relationship between provider continuity in ambulatory care and admissions	Scientific Research Institute of the regional health insurance funds ‘Allgemeine Ortskrankenkassen’ (AOKs)	382,118 heart failure patients (age ≥ 35 years)	2009-2011	COCI; UPC; SECON	9/9	Over 2009 -2010 before hospitalization	Partly, reversed causality accounted in design	Hospitalizations	Higher COC among GP, internists and cardiologists was associated with a reduction in the risk of hospitalizations (COCI: OR=0.860, CI: 0.800-0.926, UPC: OR=0.834, CI: 0.758-0.918, SECON: OR=0.752, CI: 0.692-0.818). Higher COC with GPs only was associated with lower hospitalization using SECON (OR=0.874, CI: 0.799-0.957) but not the other COC indices
Wang 2020 ;Taiwan [26]	RC	To investigate the relationships between COC and chronic conditions, how it impacted the number of outpatient visits, and risk factors of highly frequent uses	National Health Insurance Research Database (NHIRD) in Taiwan	33,294 patients who had at least one internal medicine outpatient visit	2007-2009	COCI	8/9	Single year	Partly, reversed causality accounted in design	Risk of high medical utilization (>51 visits)	Patients in the low and moderate COCI groups had a significantly higher risk of the use than did the patients in the high COCI group (OR = 2.38, CI: 2.12–2.68 and OR = 1.96, CI: 1.74–2.21, respectively). Those who had severe comorbidities (OR = 3.03, CI: 2.84–3.23) were more likely to use outpatient care highly frequently.
Worrall 2011 ; Canada [76]	RC	To examine the relationship between continuity of family physician care and all-cause mortality and acute hospitalizations	Sample from Newfoundland and Labrador portion of the National Diabetes Surveillance System (NDSS) database linked to provincial FFS physician billing database	305 older people with DM (age ≥ 65 years)	1998-1999	UPC	7/9	Over 3 years	No	Death and hospitalization rate	Higher COC group had significantly lower likelihood of hospitalizations than lower COC group (54.5% and 67.5%, respectively, *p*=0.027).
Yang 2020; Taiwan [77]	RC	To investigate the association of COC index (COCI) with medical costs and inpatient days, and investigate the possible clinical characteristics affecting the outcome	Taiwan’s National Health Insurance Research Database	3234 patients aged 0 to 18 years with cerebral palsy catastrophic illness	2000-2013	COCI	8/9	Single year	No	Medical costs and the number of inpatient days over 5 years	Five-year inpatient days for a child in the low COC group were longer than for a child in the high COC group (8 days more, *p* < 0.001). Five-year medical costs for a child in the low COC group were higher than for a child in the high COCI group (US$1656 more, *p* = 0.016). For inpatient costs: US$1660 more, *p* = 0.002.

*ACSC* Hospitalizations for ambulatory care sensitive conditions, *CHF* chronic heart failure, *CI* confidence interval, *COCI* continuity of care index, *COPD* chronic obstructive pulmonary disease, *CS* cross-sectional study, *DKA* diabetic ketoacidosis, *DM* diabetes mellitus, *ED* Emergency department, *ER* emergency room, *ESRD* End-stage renal disease, *FFS* fee-for-service, *GP* general practitioner, *HI* Herfindahl–Hirschman Index (or Herfindahl index), *aHR* adjusted hazard ratio, *HR* hazard ratio, *ICOC* Integral Continuity of Care, *LTC* long-term care, *MCI* modified continuity index, *MFPC* Most Frequent Provider Continuity, *MMCI* Modified Modified Continuity Index, *NHI* National health institute, *NR* not reported, *OR* odds ratio, *PCP* primary care physician, *PIM* potentially inappropriate medication, *RC* retrospective cohort, *SECON* Sequential Continuity, *UK* United Kingdom, *USA* United states of America, *UPC* Usual Provider Continuity, *VA* Veterans Affairs

### Characteristics of the COC measures

Overall, 14 different measures of COC were applied to claims data in the primary studies investigating associations between COC, healthcare use and costs. Among those, 12 were indices (i.e. composite measures accumulating information from various individual items) (Table 2), and two measures were non-index (Majority-of-Care Rule and Predominant Provider), used more as a reflection for provider attribution [20, 40, 68].

**Table 2:** COC index measures used in the studies to investigate associations between COC, healthcare use and costs

**Measure**	**Description**	**Range**	Concept *	**Formula**	**Interpretation**	**Number of papers using this index**
1.Bice & Boxerman index (COCI)	the degree to which patient visits are distributed among different physicians	0 (all visits to different providers) to 1 (all visits to the same provider)	dispersion^a^ density^b^	$\large \frac{{\sum }_{i=1}^{M}{n}_{i}^{2}-N}{N(N-1)}$	*N*-total number of visits, *n*_*i*_ number of visits with provider *i*, *M*-total number of providers	35
2.Usual Provider of Care (UPC)	the proportion of visits with a usual provider	0 (no visits to the usual provider) to 1 (all visits to the usual provider)	density	$\large \frac{{{max}_{i=1,\dots M} n}_{i}}{N}$	*N*- total number of visits*, n*_*i*_ – number of visits with provider *i, M-*total number of providers	14
3.Most Frequent Provider Continuity (MFPC) (similar to UPC)**	defines the primary provider as the one seen most frequently	0 (no visits to the provider) to 1 (all visits to this providermost frequent provider)	density	$\large \frac{max({n}_{1},{n}_{2}\dots , {n}_{M})}{N}$	*N* - total number of visits, *n*_*i*_ - number of visits with provider *i (i=1,…M), M-*total number of providers	2
4.Modified, Modified Continuity Index (MMCI)	the extent to which a patient concentrated her/his visits with the same healthcare provider	0 (all visits to different providers) to 1 (all visits are to the same provider)	dispersion	$\large \frac{1-\frac{M}{N+0.1}}{1-\frac{1}{N+0.1}}$	*M* -number of different providers and *N-* total number of visits.	5
5.Sequential Continuity of Care index (SECON)	the proportion of **sequential visits** that were with the same provider, i.e. same provider being seen at both the previous and current visits	0 (no sequential visits to the same provider) to 1 (all sequential visits to the same provider)	sequential ^c^	$\large \frac{{\sum }_{j=1}^{n-1}{\text{c}}_{\text{j}}}{n-1}$	if the visit *j* and the subsequent visit (*j* + 1) are to the same provider then *c*_*j*_ = 1, and *c*_*j*_ = 0 if otherwise; *n*-1- sequential pairs of visits	5
6.Herfindahl Index	indicator of provider concentration, physician’s share of a patient’s visits.	0 (less-dominant providers, not concentrated) to 1 (all visits to the same provider, very concentrated)	concentration^d^	$\large {\sum }_{i=1}^{M}{\left(\frac{{n}_{i}}{N}\right)}^{2}$	n_*i*_ - visits for patients to an individual provider *i*, and N - total number of visits, M-total number of providers	1
7.Care density	reflect how frequently patient’s doctors collaborate/ share patients with one another	0 (no collaboration/ no shared patients) to ∞ (extreme collaboration/ all patients of all doctors are shared)	density; level of shared patients between providers	$\large \frac{{\sum }_{i=1}^{m}{\text{w}}_{\text{p,i}}}{{\text{M}}_{\text{p}}({\text{M}}_{\text{p}}-1)/2}$	*M*_*p*_ - number of distinct doctors that patient *p* saw, *m* - total number of possible pairs of doctors, and *w*_*p,i*_ - number of shared patients for each pair of doctors. The numerator is the total number of instances of patient sharing over a time period (e.g. a year) among a patient’s doctors. The denominator is the total number of pairs of doctors for that patient.	3
8.Clinician Index	the proportion of visits with primary clinician out of all ambulatory visits	0 (no visits to the primary clinician) to 1 (all visits to the primary clinician)	density	*n*_*p*_*/N*_*a1*_	*n*_*p*_ - number of ambulatory visits to a primary clinician and *N*_*a1*_ - number of ambulatory visits in the 1^st^ year	1
9.Site Index	the proportion of visits with primary site out of all visits	0 (no visits to the primary site) to 1 (all visits to the primary site)	density	*n*_*p*_*/N*_*1*_	*n*_*p*_ - number of visits to a primary site and *N*_*1*_ - total number of visits in the 1^st^ year	1
**Compound Indices**						
10.Integrated Continuity of Care index (ICOC)	ICOC is a linear combination of three indices UPC, COC, SECON via Principal Component Analysis (PCA)	0 (no visits to the same provider) to 1 (all visits to the same provider)	dispersion; density; sequence	*ICOC=(β*_*1*_* UPC+ β*_*2*_*COC+β*_*3*_*SECON)/ (β*_*1*_*+ β*_*2*_*+ β*_*3*_*)*	*ß*_*1*_*, ß*_*2*_, and *ß*_*3*_ is the first principal component Eigenvector of the PCA result, which used the weighted means of the variables for each type of index	1
11.Composite index (COMP)	Composite index, derived by adding the score values of the four commonly used COC indices (COCI, UPC; MMCI, SECON) and dividing by four	0 (no visits to the same provider) to 1 (all visits to the same provider)	dispersion, density, sequence	*(UPC+COCI+MMCI+SECON)/4*	*UPC* - usual provider of care index, *COCI* - Bice& Boxerman index, *MMCI* - Modified modified continuity index, *SECON* - sequential continuity index	1
12.CI Alpha Index	weighted average of the concentration of providers seen and sequential continuity; KL represents Kullback-Leibler information index showing the degree of concentration relative to no concentration at all	0 (a different provider is seen for each patient visit, maximum dispersion in both provider concentration and visit sequence) to 1(the same provider is seen at every visit)	concentration and sequence	CIα = αKL* + (1 - α) SECON, *α ϵ [0, 1];* $KL= log M+ {\sum }_{i=1}^{M}\frac{{n}_{i}}{M} \times log \frac{{n}_{i}}{M}$*;* *and* $KL*=\frac{KL}{\mathit{log}M}$	*α* is a predetermined weight that is applied to both KL* and *SECON*; *M* - total number of providers and *n*_*i*_ is number of visits with provider *i*.	1

*PCA* Principal Component Analysis

^*^concept according to the typology developed by Jee and Cabana[7]

^**^indices UPC and MFPC were used as independent in the source studies

^a^The dispersion of visits among various providers

^b^The density of visits with a provider

^c^The number of handoffs of information required between providers

^d^The concentration of visits with a particular provider

The Bice & Boxerman Continuity Of Care Index (COCI), reflecting both density and dispersion, was the most commonly applied indicator, with 35 studies out of 46 using it. Among density measures, the Usual Provider of Care (UPC) index was the most commonly used and was included in 14 studies. The Modified Modified Continuity Index (MMCI), a dispersion measure of COC, was included in five studies. The Sequential Continuity of Care index (SECON), the only sequential measure, was used in five studies. COC was measured during different measurement periods: most studies (*N*=19) measured COC on a single year, 13 studies measured over multiple years during follow-up, and the rest of the studies had other measurement period strategies (Table 1).

### Outcomes associated with COC

Most studies reported on multiple outcomes (Table 3). Thus, out of 46 studies, the majority of studies (*N*=29) reported on all-cause and disease-specific hospitalizations or ED visits (*N*=19), and 14 studies investigated the association between COC and costs.

**Table 3:** Associations between outcomes and COC in the studies

Outcomes	Total number of studies	Number of studies observing significant improvement in outcomes associated with improved COC
Costs	14 [18, 20, 37, 41, 43–45, 47, 53–55, 57, 66, 77]	12 [18, 20, 37, 41, 45, 47, 53–55, 57, 66, 77]
All-cause and disease-specific hospitalizations	29 [18, 20, 27, 37, 38, 41, 42, 44–46, 48–51, 53–55, 57, 59–62, 67–69, 71, 73, 75, 76]	26 [18, 20, 27, 37, 38, 41, 45, 46, 48–51, 53–55, 57, 59–62, 67, 69, 71, 73, 75, 76]
Avoidable hospitalizations and ACSC	9 [20, 27, 42, 63–65, 68, 70, 73]	7 [20, 27, 64, 65, 68, 70, 73]
ED use	19 [18, 20, 27, 39, 40, 45–47, 51–53, 55, 56, 58, 59, 61, 62, 71, 73]	18 [18, 20, 27, 39, 40, 45–47, 51–53, 55, 56, 58, 59, 61, 62, 71]
Hospital readmissions	3 [66, 72, 74]	3 [66, 72, 74]
Outpatient visits use	1 [26]	1 [26]

Some studies reported on multiple outcomes, whereby one study found mixed results for two outcomes: all-cause hospitalizations and costs [44]

COC groups were determined in each individual study based on a numerical threshold of COC indices determined by the authors (e.g., 0.5, 0.75, <1) or based on the quartiles/terciles of COC measurement indices. Among the studies reporting the relationship between COC groups and odds of hospitalizations, ORs varied from 1.15 (sample of psychiatric children) [71] to 2.72 [49] (sample of children with asthma) for lower COC groups versus high COC groups, and from 0.07 [61] (sample of diabetes mellitus (DM) patients with heart failure) to 0.95 [18] (sample of COPD and DM older patients) for higher or perfect COC groups versus low COC groups. Among the studies reporting the relationship between COC and the odds of ED visits, ORs varied from 1.14 [71] (psychiatric children) to 2.25 [55] (sample of older patients with asthma) for lower COC and from 0.10 [61] (sample of DM patients with heart failure) to 0.94 [18] (sample of patients with chronic heart failure) for higher or perfect COC. Four studies out of eight focusing on avoidable hospitalization or ACSC reported the ORs, which varied from 1.12 [65] (short-term diabetes-related) to 2.29 [64] (COPD-related) for lower COC (compared to higher COC). From the studies reporting associations with costs, three reported monetary values in US dollars, whereby higher COC was associated with $1656 [77]-$4045 [43] lower total medical costs.

Whereas all studies but seven reported that higher COC was significantly associated with a reduction in healthcare use and costs, five studies found that not all tested associations were significant [42, 63, 68, 73, 75]. Specifically, the following associations did not reach significance: low COC group in short-term with risk of avoidable hospitalization (OR=1.59, CI: 0.91–2.76) [63]; high COC with all-cause hospitalizations (OR = 0.83, CI: 0.67–1.01) [68]; high COC with ED visits (OR=2.6, CI: -0.2–5.4) [73]; high COC with general practitioner (GP) measured using COCI (OR=0.953, CI: 0.884–1.029) and UPC (OR=0.940, CI: 0.849–1.040), in contrast to SECON, with risk of hospitalizations; high COC with ACSC hospitalizations (β=-0.2, CI: -2.1–1.8) [42]. Moreover, one study found unexpected direction of association for a secondary outcome [43], whereby higher COC increased costs by $402 for medical long-term care and by $764 for social long-term care. Finally, one study found mixed evidence on the associations for both tested outcomes (risk of hospitalization and costs), depending on the choice of COC index and type of consultations [44].

Due to substantial heterogeneity of COC and healthcare use measurements and statistical methods applied, it appeared infeasible to pool the studies to produce an estimate of effect size. Details of statistical metrics of individual studies are available in Table 1.

## Discussion

Currently, several existing reviews focus on various aspects of COC [4, 6, 14, 78], existing COC measures [7, 8, 79, 80] and associations between COC and various health outcomes [21–23, 81]. Our review is the first to narrow down the focus to a specific list of longitudinal COC measures, used only with claims-based data to examine the relationship between longitudinal COC and specific outcomes: healthcare utilization and costs. We identified 46 primary studies conducted mainly in North America and East Asia that considered 14 COC indicators applied to claims-based data. Of those indicators, the COCI and UPC index, representing the concepts of dispersion and density, respectively, were the most commonly used indices. Our results also show that higher COC was frequently associated with decreased healthcare costs and utilization.

The UPC index is relatively easy to calculate and interpret, which might explain the frequency of its use. However, this measure does neither take into account the number of providers seen, nor the distribution of visits to other providers, posing a challenge for chronic patients who need specialty care from “several” providers other than the “usual” one. COCI, in contrast, considers the aforementioned aspects, but is more cumbersome to compute and overly sensitive to increasing number of providers, which leads to likely flawed conclusions about COC for patients with chronic conditions.

In fact, the main issue of currently existing COC indices applied in claims data is their inability to fully capture the multidimensional construct of COC. Longitudinal continuity is usually used in claims-based studies to exhibit interpersonal continuity, as it was assumed that recorded repeated contacts between a patient and care provider already represented a reliant and stable relationship [23]. As all the studies in the review relied on claims data, they focused on longitudinal dimension of continuity, while being unable to potentially integrate other dimensions of COC (e.g., interpersonal or informational). This leaves the gaps in assessing the effect of information or interpersonal continuity on health outcomes. It can be argued that information continuity is likely to be improved more easily than longitudinal continuity from operational perspective, by the implementation of information systems that make patient information more available to all health care providers [20]. Therefore, future studies should try find ways of incorporating broader aspects of COC, such as information exchange, management structure, or interpersonal relations. Moreover, the mentioned above indices do not capture the appropriateness of care, which is important for accurate interpretation of COC and assessment of the need for better COC, especially for patients with chronic conditions [82]. Whereas it may be appropriate to only visit a family doctor and no specialists, chronic care may often imply appropriately visiting several providers, which could result in low COC measures.

Measuring the impact of continuity on healthcare outcomes and costs is rising in the literature [83], albeit the standing aforementioned limitations. In the present review, all studies clearly defined the COC measures and used claims based administrative data to report on the associations between healthcare use and costs. Higher COC was associated with lower costs or healthcare use in any age group with a specific disease or within the general population, despite using various measures of COC and different settings in, but not limited to, countries (e.g., Korea, Taiwan, Norway, and USA) with national healthcare systems and universal coverage. Moreover, all but seven studies included in this review found this association significant for all tested outcomes. Specifically, in the five studies investigating associations with multiple outcomes, the partial results did not reach significance for distinct types of secondary outcomes [42, 63, 68, 73, 75]. One study investigating the causal effect of COC on costs found that higher COC was associated with higher non-institutional cost, but the effect was counterbalanced by lower institutional care cost, resulting in lower total cost [43]. Finally, one Swiss study investigating associations between COC, hospitalizations and costs among cancer patients found highly mixed results, whereby the type of COC index used and the type of consultation highly influenced the results [44]. It needs to be emphasized that the results of the aforementioned studies diverted only partially from the general findings, which suggested overall robust associations between COC and healthcare use and costs. Thus, our review shed light on an emerging consensus in terms of the direction of associations between COC and outcomes that were commonly identified in claims-based studies on healthcare use and cost. These findings emphasize the need to foster COC and to develop continuity-improving strategies, which may be potentially considered for future research: gatekeeping or managed care healthcare models, financing mechanisms for healthcare providers, data sharing and incentives for care coordination and professional collaboration [12, 18, 48, 72, 84–86].

### Strengths and limitations of the present study

Our study has two main strengths. First, we focused on claims data that are relatively easily accessible and routinely collected on a large scale by public and private entities in many healthcare systems. One key advantage of claims-based data is that in most instances the unit of observation is the enrolled individual and not the provider, so they are more efficient at capturing the journey of patients across providers, whereas provider-based administrative data (e.g., Hospital Episodes Statistics in the UK) do not allow for its own the study of continuity. Second, we comprehensively presented the currently used COC indicators in the published literature, and shed light on the existing studies showing impact of COC on healthcare use and costs in various patients’ and country settings.

Nevertheless, we acknowledge two main limitations in our study. First, the fact that we performed a rapid systematic review, whereby some steps or components of systematic review may be simplified; in our case, it concerned the selection, data extraction and verification phases, which were not performed by two reviewers at all stages. For transparency of our approach, we published our protocol in PROSPERO [33]. Second, the fact that our study may be prone to biases and shortcomings of claims-based data studies. For instance, we did not have details of clinical information, diagnosis details, or use of over-the-counter medications that were not covered/collected by the insurance system, which makes the estimation of associations between COC measures and healthcare costs and use less accurate. It is important to emphasize that the way the providers are reimbursed impacts data collection: in fee-for-service systems, patient-physician interactions are observed whenever patients see their medical doctors, but in global budgets systems, we might not see these interactions, or underestimate them. Additionally, causality, associated time-bias and omitted variables bias apply to all the evidence presented in this review. Most of the studies either acknowledged inability to correct for these issues in the limitations, or adjusted their design and analysis by introducing a lag for the measurement of COC and outcomes, or including time-dependent variable in COC modeling. However, accounting for time-bias is not sufficient to make claims of causal inference, which requires dedicated study design and modeling techniques. Finally, most of the indices were measured in the short-term (1-2 years), while applying a longer observation period could be appropriate to assess whether sustained high COC is associated with health outcomes, especially since the richness of claims data allows for measurements in the long-term.

## Conclusions

Despite a variety of currently used COC indicators, it remains difficult to find a measure that fully captures the multidimensionality of continuity. Although all used measures have drawbacks and challenges for estimating COC in chronic patients, our study on the association of COC with health outcomes shows that higher COC is associated with lower healthcare costs and use, which holds true for various countries with distinct healthcare systems. These results highlight the need of an effective healthcare delivery system promoting COC, as it is an important factor in managing diseases to reach more favorable health outcomes with lower healthcare expenses. As COC is a multifaceted construct, policy makers should obtain evidence not only on the longitudinal COC, covered in the current review, but also informational (e.g., electronic health records promotion and effective management) and interpersonal COC (established patient-provider relationships). Future studies should incorporate multiple aspects of COC to cover a broader picture, by making use of patient satisfaction surveys or healthcare pathways information, and cover the causality issue that was found to be problematic in many of the reviewed studies, and apply a design allowing for causal inference.

## Supplementary Information

**Additional file 1.**

## Data Availability

Data sharing not applicable to this article as no datasets were generated or analysed during the current study.
