# Supplementary material for: Association between continuity of care (COC), healthcare use and costs: what can we learn from claims data? A rapid review
Source: BMC Health Serv Res. 2022 May 16;22:658. doi: 10.1186/s12913-022-07953-z (PMC9112559; doi:10.1186/s12913-022-07953-z)
Supplement: Supplementary file 1 — Additional file 1. [file 12913_2022_7953_MOESM1_ESM.pdf]

Medline via Pubmed (https://pubmed.ncbi.nlm.nih.gov)

"Continuity of Patient Care"[Mesh] OR “continuity” [tiab] OR collaborat* [tiab] OR communic* [tiab] OR coord* [tiab] OR care integration [tiab] AND (“index” [tiab] OR indic* [tiab] OR “measur*” [tiab] OR “indicator*” [tiab]) AND ("Insurance Claim Review"[MeSH] OR "claims-based data"[tiab] OR "claims data"[tiab] OR "billing data"[tiab] OR "hospitalisation data"[tiab] OR "hospitalization data"[tiab] OR insurance claim*[tiab] OR "insurance data"[tiab])

Output: 580 (date of initial search: 01 April 2019)

Embase (www.embase.com)

History Save | Delete | Print view | Export | Email Combine > using And Or ^ Collapse

History		
#19	#16 AND #17 AND [1-1-1966]/sd NOT [2-4-2019]/sd	942
#18	#16 AND #17	1,364
#17	#14 AND #15	501,517
#16	#10 OR #11 OR #12 OR #13	29,038
#15	#7 OR #8 OR #9	9,240,678
#14	#1 OR #2 OR #3 OR #4 OR #5 OR #6	1,854,049
#13	insurance NEAR/2 claim*	7,094
#12	claim* NEAR/2 data	21,191
#11	insurance NEAR/2 data	4,352
#10	'billing and claims'/exp	1,991
#9	measur*:ab,ti	4,904,421
#8	indic*:ab,ti	4,551,159
#7	index*:ab,ti	1,337,695
#6	integration NEAR/2 care	1,917
#5	coord*:ab,ti	332,899
#4	communic*:ab,ti	454,286
#3	collaborat*:ab,ti	239,636
#2	continuity:ab,ti	50,135
#1	'continuity of patient care'/exp	913,460

Cochrane library (https://www.cochranelibrary.com)

View fewer lines Print

#1	(Continuity):ti,ab,kw	Limits	2616
#2	collaborat*:ab,ti	Limits	12971
#3	communic*:ab,ti	Limits	23008
#4	coord*:ab,ti	Limits	11350
#5	integration NEAR/2 care	Limits	141
#6	Index*:ab,ti	Limits	139620
#7	indic*:ab,ti	Limits	181394
#8	measur*:ab,ti	Limits	476687
#9	'billing and claims'	Limits	51
#10	Claims data	Limits	2901
#11	Any MeSH descriptor in all MeSH products	MeSH	0
#12	Insurance Claim Reporting	Limits	52
#13	Continuity of Patient Care	Limits	1634
#14	#13 OR #12 OR #1 OR #2 OR #3 OR #4 OR #5	Limits	47117
#15	#6 OR #7 OR #8	Limits	655598
#16	#9 OR #10	Limits	2901
#17	#14 AND #15 AND #16	Limits	139

with Cochrane Library publication date from Jan 1900 to Apr 2019
